# Association News

**Published:** 1900-03

**Authors:** 


### ASSOCIATION NEWS.

THE February meeting of the Keystone Veterinary Medical Association, which was devoted entirely to a consideration of the milk problem and addressed by a number of experts who have given the subject special attention from the various points of view, will be fruitful of much good in the better direction of this work in Philadelphia and many other municipalities.

The Genesee Valley Veterinary Medical Association was quick to realize the danger to the Empire State livestock industry without the same protection as Pennsylvania, Massachusetts, Maine, and other States, and have earnestly and aggressively advocated protective legislation preventing the importation of cattle into the States that are not known to be free from tuberculosis. The authorities in New York State will do well to pay heed to this advice, for nothing threatens greater danger and injury to her dairy interests than the continuance of the open-door policy to the perpetuation of this white plague.

The Minnesota State Veterinary Medical Association recently held a record-breaking meeting in point of attendance and gained eight new members.

---

